# Fatty acid biosynthesis and transcriptional regulation of Stearoyl-CoA Desaturase 1 (SCD1) in buffalo milk

**DOI:** 10.1186/s12863-020-0829-6

**Published:** 2020-03-02

**Authors:** Zhipeng Li, Suyu Lu, Kuiqing Cui, Laiba Shafique, Saif ur Rehman, Chan Luo, Zhiqiang Wang, Jue Ruan, Qian Qian, Qingyou Liu

**Affiliations:** 1 Agricultural Genomics Institute, Chinese Academy of Agricultural Sciences, Shenzhen, 518120 Guangdong China; 2 State Key Laboratory for Conservation and Utilization of Subtropical Agro-Bioresources, Guangxi University, Nanning, 530005 Guangxi China

**Keywords:** SCD1, Buffalo milk, Fatty acid, RNA interference, Overexpression

## Abstract

**Background:**

Buffalo milk is considered as a highly nutritious food owing to its higher contents of fatty acids (FA) and rich nutrient profile. Higher fat contents of buffalo milk make it suitable for processing to develop various healthy and nutritious products. Moreover, buffalo milk contains more unsaturated FAs (UFA) such as oleic and linolenic acid, which are important from the human health point of view owing to their desirable physiological effects. However, inadequate information is available about the chemical composition and mechanism of FA synthesis in buffalo milk. In this study, we hypothesized that expression of *SCD*1 gene could alter the biosynthesis of FA in epithelial cells of mammary gland and subsequently affect the FA contents in buffalo milk. We investigated the transcriptional and biological role of Stearoyl-CoA Desaturase 1 (*SCD*1) in the buffalo mammary epithelial cells (BMECs) during FA and triacylglycerol (TAG) synthesis.

**Results:**

Results revealed that unsaturated fatty acid contents were much higher in concentration in buffalo milk as compared to Holstein cow. Significant increase in the expression level of *FAS*, *ACACA*, *SREBP*1, *PPARG*, *GPAT,* and *AGPAT* genes was observed in response to altered expression of SCD*1* in buffalo milk. Moreover, change in *SCD1* gene in BMECs also mediated the expression of genes related to FA biosynthesis subsequently leading to alter the FA composition. Overexpression of *SCD1* significantly increased the expression of genes associated with FA and TAG synthesis leading to enhance FA and unsaturated FA contents in BMECs. However, down-regulation of *SCD1* exhibited opposite consequences.

**Conclusion:**

Our study provides mechanistic insights on transcriptional regulation of *SCD*1 to alter FA and TAG synthesis through directly or indirectly mediating biosynthesis and metabolic pathways in BMECs. We provide preliminary findings regarding engineering of FA contents in buffalo milk through *SCD*1 signaling.

## Background

Buffalo milk is the second most-produced milk in the world after cow milk and especially important in the Asian continent which inhabits more than 97% of the global buffalo population [1, 2]. Buffalo milk is a rich source of fatty acids (FA) with high energetic and nutritive value. Higher fat contents of buffalo milk make it suitable for processing to develop various delicious, healthy and nutritious products. Moreover, Buffalo milk contains more unsaturated FAs (UFA) such as oleic and linolenic acid, which are important from human health point of view owing to their number of desirable physiological effects [3, 4]. However, inadequate information is available about the chemical composition and mechanism of FA biosynthesis in buffalo milk.

Milk FAs are either de novo synthesized in the mammary gland or imported from the plasma. Signaling mechanisms of lipogenesis have been clearly described in rodent tissues [5], but limited studies are available in buffalo regarding this. However, studies have suggested that fat synthesis in the mammary gland may share a similar mechanism [6, 7]. It has been also observed that main lipid metabolic pathways are controlled by DNA-binding activity and nuclear abundance of selected transcription factors of key regulatory genes [5, 8]. Major transcriptional factors involved in FA biosynthesis are sterol regulatory binding protein-1 (SREBP-1) and peroxisome proliferator-activated receptors (PPARs). FA or cholesterol can induce changes in the nuclear abundance of SREBP and bind to the nuclear receptors (PPAR). SREBP can bind with sterol binding elements or palindromic sequences on the promoter regions of its target genes such as acetyl-CoA carboxylase (*ACACA*), FA synthase (*FAS*), and Stearoyl-CoA Desaturase (*SCD*) [8]. An implication of PPARG in nutritional or insulin activation of lipogenesis has been observed by upregulation of *PPARG*, *FAS* and *ACACA* gene expression in bovine tissues [9]. The acetyl-CoA carboxylase (ACC) and FA synthase (FAS) enzymes (encoded by the *ACACA* and *FAS* genes, respectively) are involved in the metabolic pathway for de novo FA synthesis [8]. The ACCA protein provides cytoplasmic malonyl-CoA for FA synthesis while FAS protein is mainly responsible for the synthesis of short- and medium-chain FA (C4-C16) [10].

In the mammary gland, *SCD* is responsible for the synthesis of the major part of cis-9, trans-11- [11, 12] and of trans-7, cis-9 conjugated linoleic acids (CLA) [13]. These FAs can be desaturated by *SCD* leading to the synthesis of cis-9 monounsaturated FA (MUFA), which are then esterified to glycerol sequentially by glycerol-3 phosphate acyl transferase (GPAT), acyl glycerol phosphate acyl transferase (AGPAT), and diacylglycerol acyl transferase (DGAT) [8]. The promoter region of bovine SCD, especially the region containing the stearoyl-CoA desaturase transcriptional enhancer element, plays a key role in the inhibition of transcriptional activity of trans-10, cis-12 CLA. It is recognized as a core gene involved in the FA and triacylglycerol (TAG) synthesis [14] and potential tool to study transcriptional regulation of milk quality.

Several studies have reported FA composition and physico-chemical characteristics of buffalo and cow milk; however, few studies have focused on illustration of molecular mechanisms. Therefore, present study was conducted to testify the hypothesis that transcriptional and biological role of SCD1 (during FA and TAG synthesis) affects the FA contents of Buffalo milk. Initially, we determined the FA composition of buffalo and cow milk and analyzed the expression pattern of the respective genes related to FA synthesis. Later on, association of *SCD1* with selected genes was further studied by RNA interference (RNAi) and overexpression of *SCD1* in Buffalo mammary epithelial cells (BMECs), to elucidate potential effects on FA compositions. Transcriptional regulation of SCD1 could be used to provide mechanistic insights on physico-chemical characteristics of buffalo milk.

## Results

### Routine analysis of Milk composition

Chemical composition of Holstein cow and buffalo milk was evaluated including, butter fat, protein, lactose, and total solid and solid not fat contents (Table 1). Results revealed that lactose contents were comparable in cow and buffalo milk, while FA, protein, total solids and solid not fat contents were significantly higher in buffalo as compared to cow milk. The Highest difference was observed in butterfat contents which were 1.86 times higher in buffalo than cow milk (7.88 ± 0.91 vs 4.24 ± 0.80).

**Table 1:** Routine composition of milk in different cows

Species	Fat %	Protein %	Lactose %	Total solids %	Solid not fat %
Holstein	4.24 ± 0.80^a^	3.39 ± 0.55^a^	4.92 ± 0.32	13.40 ± 1.36^a^	9.17 ± 0.58^a^
Buffalo	7.88 ± 0.91^b^	5.37 ± 0.32^b^	4.99 ± 0.41	18.16 ± 1.08^b^	10.23 ± 0.47^b^

Note: Different superscript letters means significantly different (*P* < 0.05)

### Analysis of FA composition of Milk

Gas chromatography was used to analyze the FA composition of milk samples (Table 2). Results showed that FA contents of milk from both species were similar, except CLA and Eicosapentaenoic acid (EPA) which were only observed in buffalo milk. The C16:0 was major saturated FA (SFA) while C18:1 was major unsaturated FA (UFA) in milk as observed in our study. Each kind of FA and the total FA in Buffalo milk are all significantly richer than that of Holstein cows in terms of the FA content per 100 g of milk (*p* < 0.05).

**Table 2:** FA composition of Holstein and buffalo milk

Fat acid		Holstein	Buffalo
Saturated Fat acid	C4:0	36.96 ± 10.92 ^a^	94.49 ± 13.39 ^b^
	C6:0	20.09 ± 3.75 ^a^	116.2 ± 9.98 ^b^
	C8:0	13.59 ± 3 ^a^	73 ± 6.41 ^b^
	C10:0	24.72 ± 4.41 ^a^	151.27 ± 11.23 ^b^
	C12:0	33.82 ± 6.2 ^a^	183.52 ± 16.03 ^b^
	C14:0	105.2 ± 19.7 ^a^	854.22 ± 58.83 ^b^
	C15:0	0.94 ± 0.17 ^a^	10 ± 1.27 ^b^
	**C16:0**	**213.36 ± 36.49** ^**a**^	**2986.31 ± 178.62** ^**b**^
	C17:0	7.37 ± 1.74 ^a^	81.52 ± 8.62 ^b^
	C18:0	51.77 ± 8.47 ^a^	957.3 ± 114.08 ^b^
	Total	507.82 ± 94.85 ^a^	5507.83 ± 418.46 ^b^
Unsaturated Fat acid	C14:1	11.55 ± 1.76 ^a^	92.23 ± 10.58 ^b^
	C16:1	22.06 ± 4.7 ^a^	247.68 ± 33.77 ^b^
	C17:1	2.89 ± 0.53 ^a^	29.58 ± 3.2 ^b^
	**C18:1**	**343.33 ± 62.99** ^**a**^	**2419.31 ± 257.48** ^**b**^
	C18:2	28.97 ± 5.88 ^a^	136.34 ± 15.24 ^b^
	C18:3	5.76 ± 1.49 ^a^	40.14 ± 4.89 ^b^
	C20:1	1.89 ± 0.53 ^a^	23.25 ± 3.64 ^b^
	C20:2	1.2 ± 0.17 ^a^	3.62 ± 0.45 ^b^
	C20:3	3.15 ± 0.59 ^a^	14.21 ± 1.39 ^b^
	**CLA**	**–**	**19.4 ± 1.68**^**a**^
	**EPA**	**–**	**14.6 ± 0.2**^**a**^
	Total	449.45 ± 78.62 ^a^	2959.94 ± 327.72^a^
Total		925.27 ± 173.47^a^	8467.77 ± 746.18^b^

Note: “—” indicates undetectable; Different superscript letters mean significantly different (*P* < 0.05). All milk FA compositions were expressed as mg/100 g of fat

### Expression of genes related to FA synthesis in Milk

**Fig. 1:**
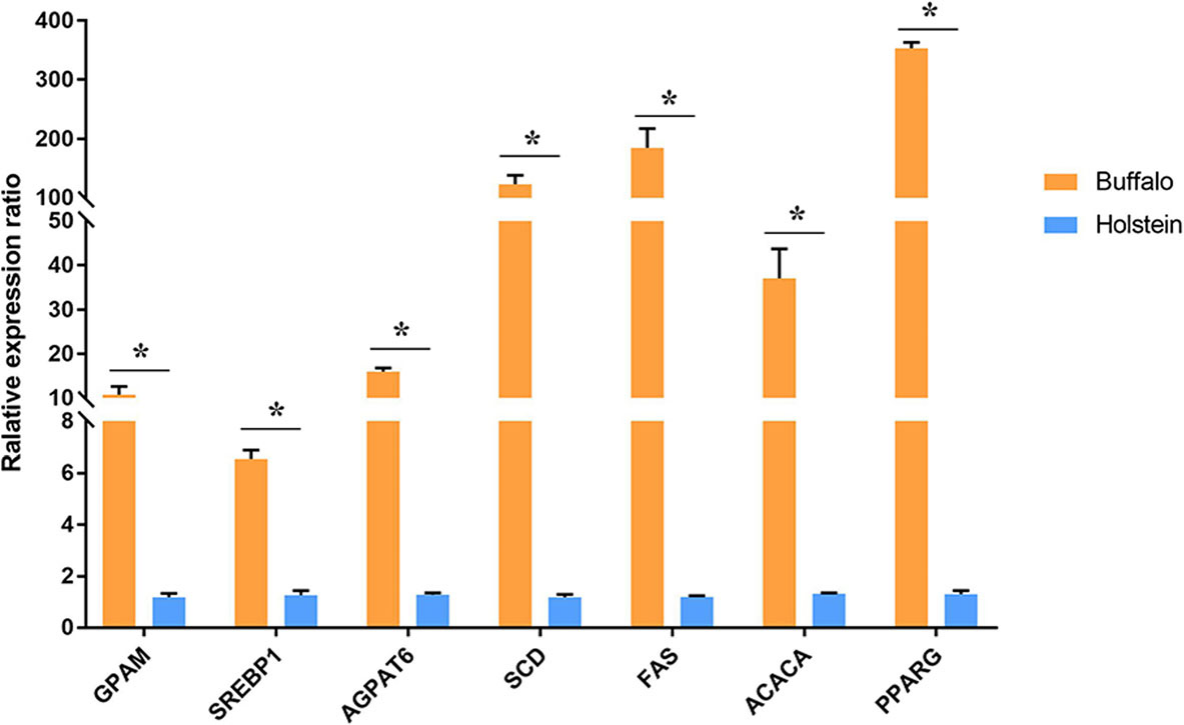
Expression of genes related to FA synthesis in milk by qRT-PCR analysis. Note: Different superscript letters means significantly different (*P* < 0.05)

Expression of key genes related to milk fat synthesis in both species was detected by QRT- PCR. Results revealed radically higher expression level of genes related with fat synthesis including FA de novo synthesis related genes (*FAS* and *ACACA)*, Glyceride synthesis related genes (*GPAT* and *AGPAT*6), and transcriptional regulator related genes (*SREBP*1 and *PPARG)* in buffalo as compared to cow milk (Fig. 1). In addition, the expression of *SCD*1 in buffalo milk was about 123 times higher than that of cow, which indicates its key role in FA synthesis in milk.

### Effect of SCD1 interference on expression of FA synthesis related genes and subsequent FA composition in BMECs

**Fig. 2:**
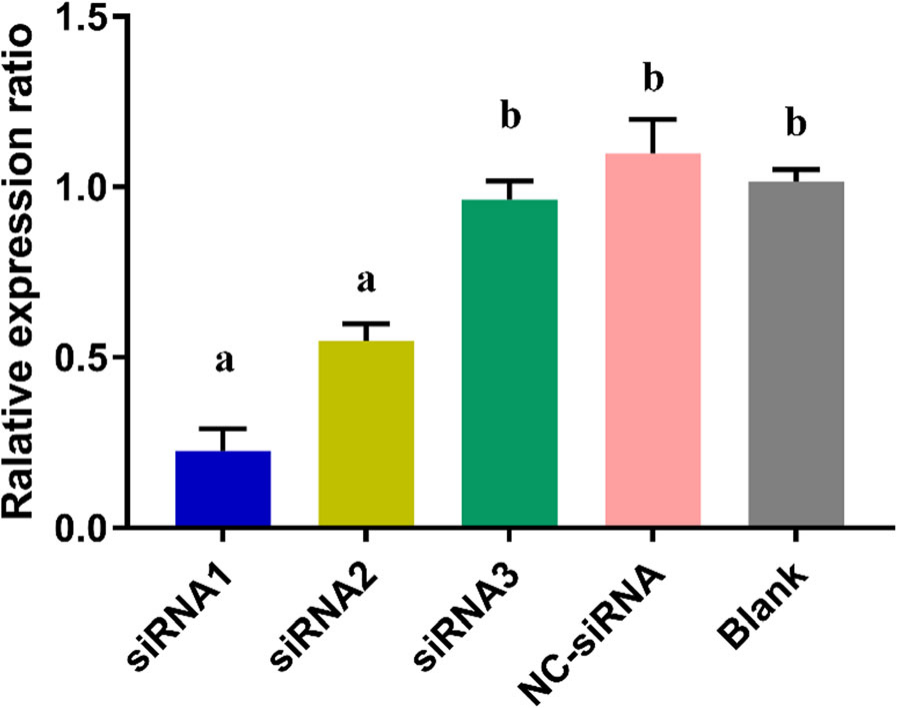
The relative expression of SCD1 after siRNA interference. Note: Different superscript letters means significantly different (*P* < 0.05)

**Fig. 3:**
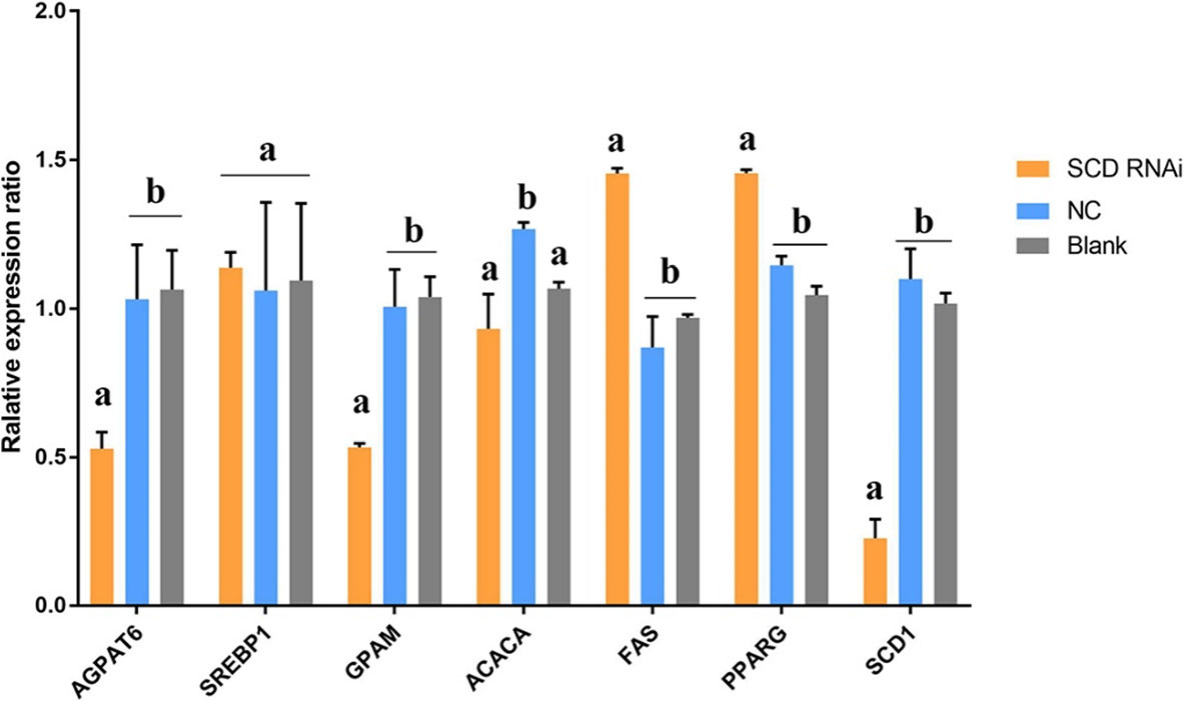
Expression of FA synthesis related genes after SCD1 interference. Note: Different superscript letters means significantly different (*P* < 0.05)

The interference efficiency of the siRNA was detected first to confirm the feasibility of the siRNA. Results indicated that the transfection of siRNA1 and siRNA2 significantly decreased the expression of *SCD1* as compared to the control group, while siRNA3 and NC-siRNA exhibited no significant effect on expression level (Fig. 2). Keeping in view the greater potential of siRNA1 to interfere the expression of *SCD* gene, it was selected for further study. After the transfection of BMECs with siRNA1 and NC-siRNA respectively, the expression of FA synthesis related genes was determined (Fig. 3). Results demonstrated that the expression of *SCD*1 was significantly decreased (1/5th) as compared to blank control, indicating successful siRNA transfection. Expression of *FAS* and *PPARG* considerably increased (*P* < 0.05), whereas expression of *AGPAT*6, *ACACA,* and *GPAT* gene decreased (*P* < 0.05) after interference. However, *SCD1* interfering did not significantly affect the expression of *SREBP*1 (*P* > 0.05).

The FA contents extracted from cells were further analyzed by gas chromatography (Table 3). Results demonstrated that siRNA interference significantly increased (*P* < 0.05) SFA contents (C16:0 and C18:0), while decreasing (P < 0.05) UFA contents (C16:1 and C18:1). Moreover, total SFA contents also significantly increased (P < 0.05) while total UFA markedly decreased (P < 0.05). Interestingly, no significant change (*P* > 0.05) was observed in the total FA contents of the BMECs.

**Table 3:** Effect of SCD1 interference on FA composition in BMECs

Fat Acid	Blank control (mg)	Negative control (mg)	RNA interfered (mg)
C16:0	0.614 ± 0.11 ^a^	0.591 ± 0.11 ^a^	0.759 ± 0.18 ^b^↑
C16:1	0.279 ± 0.06^a^	0.226 ± 0.03 ^a^	0.124 ± 0.03^b^↓
C18:0	0.657 ± 0.16 ^a^	0.547 ± 0.07 ^a^	0.875 ± 0.17 ^b^↑
C18:1	0.873 ± 0.10 ^a^	0.886 ± 0.18 ^a^	0.613 ± 0.15 ^b^↓
C18:2	0.212 ± 0.06	0.203 ± 0.02	0.216 ± 0.02
Total SFA	1.271 ± 0.27 ^a^	1.138 ± 0.17 ^a^	1.634 ± 0.36 ^b^↑
Total UFA	1.364 ± 0.22 ^a^	1.315 ± 0.24 ^a^	0.953 ± 0.20 ^b^↓
Total	2.535 ± 0.49	2.453 ± 0.41	2.587 ± 0.56

Note: Different superscript letters means significantly different (*P* < 0.05). All milk FA compositions were expressed as mg/100 g of fat. *SFA* Saturated Fat acid, *UFA* Unsaturated Fat acid

### Effect of SCD1 overexpression on FA synthesis related genes and FA composition in BMECs

**Fig. 4:**
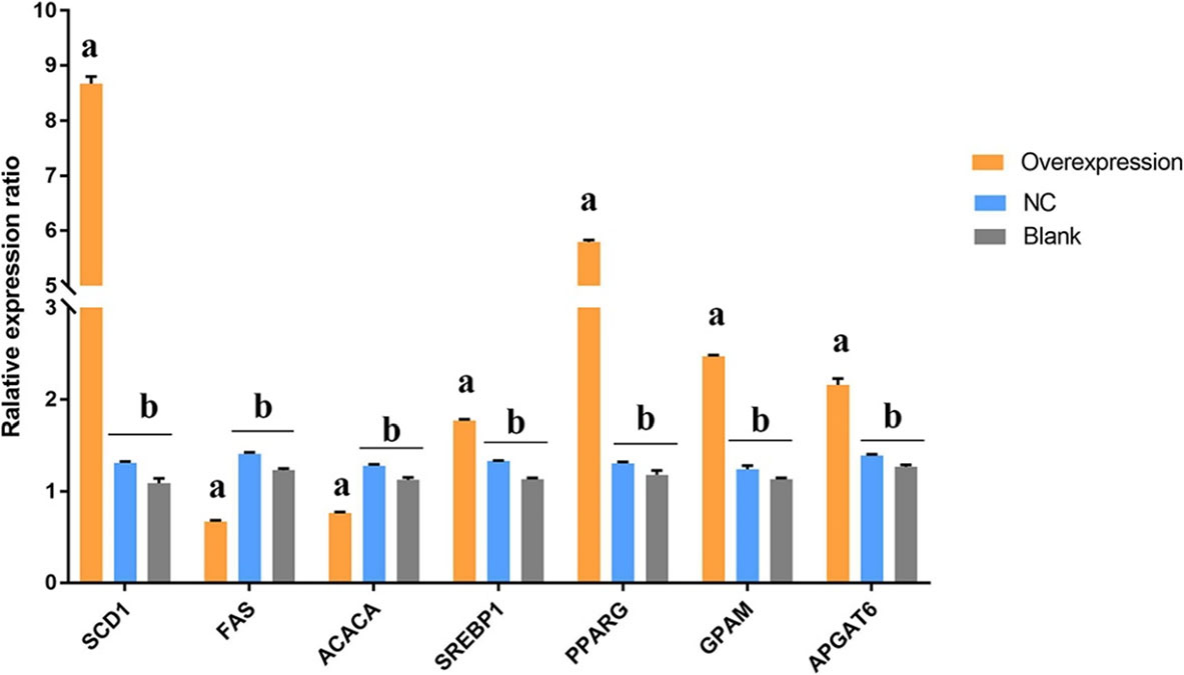
Expression of FA synthesis related genes after SCD1 overexpression. Note: Different superscript letters means significantly different (*P* < 0.05)

After SCD1 overexpression, the expression of SCD1 increased up to 8 times more as compared to the blank control. The expression level of both FAS and ACACA was significantly downregulated, whereas expression of GPAT, SREBP1, PPARG and AGPAT was significantly enhanced (Fig. 4). The composition of FA that extracted from BMECs was also analyzed by applying Gas chromatography (Table 4). Overexpression of SCD1 in BMECs resulted in a significant (*P* < 0.05) decrease in SFA contents (C16:0 and C18:0) while increasing UFA (C16:1, C18:1 and C18:2) contents. Moreover, total FAs and UFA contents in the cells were increased significantly (P < 0.05), while total SFA contents were decreased (*P* < 0.05).

**Table 4:** Effect of SCD1 overexpression on FA composition in BMECs

Fat Acid	Empty control (mg)	Negative control (mg)	Overexpressed (mg)
C16:0	0.614 ± 0.11 ^a^	0.602 ± 0.09 ^a^	0.403 ± 0.10 ^b^↓
C16:1	0.279 ± 0.05 ^a^	0.256 ± 0.06 ^a^	0.486 ± 0.09 ^b^↑
C18:0	0.657 ± 0.16 ^a^	0.642 ± 0.12 ^a^	0.319 ± 0.05 ^b^↓
C18:1	0.873 ± 0.11 ^a^	0.821 ± 0.15 ^a^	1.276 ± 0.17 ^b^↑
C18:2	0.212 ± 0.06 ^a^	0.197 ± 0.06 ^a^	0.354 ± 0.03 ^b^↑
Total SFA	1.271 ± 0.27 ^a^	1.244 ± 0.22 ^a^	0.722 ± 0.16 ^b^↓
Total UFA	1.364 ± 0.22 ^a^	1.274 ± 0.27 ^a^	2.116 ± 0.29 ^b^↑
Total	2.535 ± 0.50 ^a^	2.518 ± 0.48 ^a^	2.838 ± 0.45 ^b^↑

Note: Different superscript letters means significantly different (*P* < 0.05). *SFA* Saturated Fat acid, *UFA* Unsaturated Fat acid

### Summary of gene networks involved in Milk fat synthesis

**Fig. 5:**
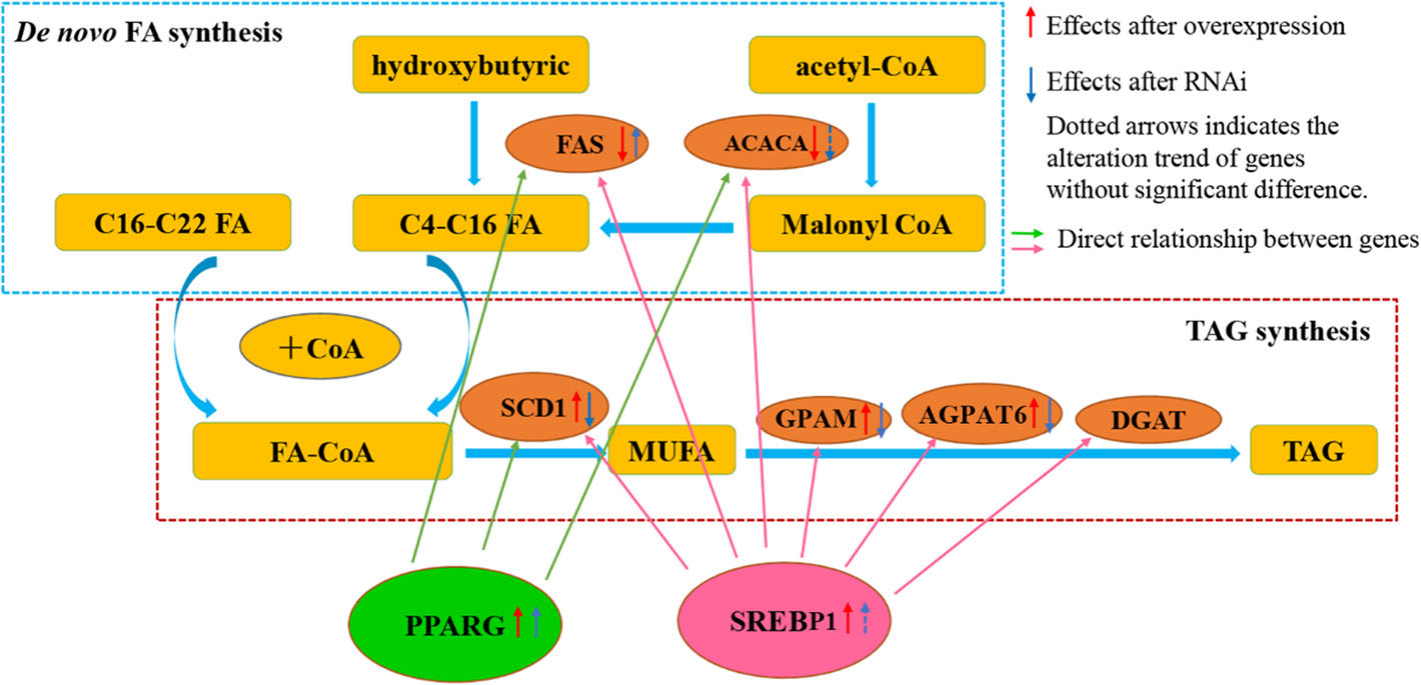
Summary of SCD1 associated FA synthesis and metabolism signal pathways in BMECs

The networks between SCD1 and genes related to FA synthesis in BMECs were analyzed (Fig. 5). Results revealed that knockdown of SCD1 can significantly decrease the expression of TAG biosynthesis related genes (*GPAT and AGPAT6*), while increasing the expression of FA synthesis genes as compared to control. Similarly, the expression of these genes showed the opposite trend when SCD1 was overexpressed, which was in accordance with the alteration of SCD1. Expression of the ACACA gene was decreased either due to SCD1 overexpression or siRNA treatment, despite the change induced by siRNA was not significant. However, expression of transcription factor PPARG was significantly up-regulated regardless of overexpression or knockdown of SCD1. Furthermore, transcription factor SREBP was significantly up-regulated in response to SCD1 overexpression, but not markedly affected by the down regulation of SCD1.

## Discussion

Milk is an important part of the human diet that provides nourishment and essential nutrients to the body. It is also relished by individuals and also used to make a variety of delicious and healthy food products. In our study, we observed significantly higher protein and FA contents (especially UFA) in buffalo milk as compared to cow milk. Similar findings have been reported earlier [1, 2]. But we observed contrary findings regarding lactose contents which are generally reported higher in buffalo milk compared to the cow [2]. This variation may be attributed to different dietary, environmental and physiological conditions of animals used in this study. However, this does not affect the buffalo milk quality and low lactose contents may be even beneficial for individuals having lactose intolerance [15].

In the present study, expression of genes responsible for FA de novo synthesis (*FAS* and *ACACA*), TAG synthesis (*GPAT* and *AGPAT*) and transcription factors (*SREBP*1 and *PPARG*) was significantly higher in buffalo than cow. These findings support the evidence that higher FA contents in buffalo milk are associated with over expression of these genes, as reported earlier in goat and bovines [16–18].

Extensive studies conducted on lactation biology of bovine mammary gland have [19].SREBPs are transcriptional factors that regulate lipid homeostasis through controlling the expression of enzymes required for FA de novo synthesis. The mature SREBP is translocated to the nucleus, where it binds with its target genes, including *ACACA*, *FAS*, *SCD*1, and others [20]. Moreover, SREBP1 is a central element in the overall regulation of genes involved in milk fat synthesis [16, 21], and SCD1 is involved in the SREBP-1-regulated pathway during milk fat synthesis [22, 23]. Two isoforms of SREBP1 protein, SREBP1a and SREBP1c, usually exist in mammals which arise by the use of two alternative first exons. Exon 1a encodes the unique 29 amino acids of the SREBP1a NH2-terminus, whereas exon 1c only encodes 5 unique amino acids [24, 25]. Experiments in transfected cells have shown that SREBP-1c is a much weaker activator of transcription than SREBP-1a when both are simultaneously expressed [26]. However, the ratio of SREBP-1c to 1a transcripts has shown remarkable variation among different organs of adult mouse and human [25]. Considering the similar function of these two isoforms in the regulation of lipid homeostasis, our study did not show any variation in the transcription of both proteins. The primer of SREBP1 used in this study was spanning the exon 2 to exon 4, to amplify both SREBP-1a and 1c. In the present study, change in mRNA levels of *SREBP*1in response to overexpression or RNAi of *SCD1* clearly demonstrated that *SREBP*1 could be regulated transcriptionally by *SCD*1 in BMECs, which is consistent with earlier studies as mentioned above. PPARs belong to a superfamily of hormone receptors and regulate transcription of genes that are involved in different metabolic pathways of lipids, including transport of plasma triglycerides, cellular uptake of FA, and peroxisomal and mitochondrial β-oxidation [27].

It has been reported that activation of *PPARG* can regulate lipogenesis and expression of *FAS* and *ACACA* [9]. Although, the activation of *PPARG* did not affect the expression of *SCD*1 in rodents [28, 29], the positive effect in ruminants has been stated earlier [18, 23, 30]. Our findings also confirmed the direct association of SCD1 with *PPARG* during FA synthesis. Furthermore, studies have also reported that expression of *SCD*1 can be suppressed by the natural ligands of *PPARG*, palmitoleic acid and oleic acid, through a polyunsaturated FA response region (PUFA-RR) [31, 32]. Therefore, an appropriate ratio of intracellular oleic to stearic acid should be maintained for proper regulation of *SCD*1, *SREBP*1 and *PPARG*.

Several studies have demonstrated a significant association of polymorphism in *SCD*1 with milk FA composition, which indicates the crucial role of *SCD*1 in mammary FA and TAG synthesis [33–35]. Although *SCD*1 is mainly involved in the synthesis of MUFA but it can also affect the synthesis of TAG. The MUFA catalyzed by SCD1 may even serve as a substrate for TAG synthesis in the mammary gland [36]. In the present study, *SCD*1 expression was associated with the expression of genes related to FA and TAG synthesis in BMECs which is in agreement with earlier studies mentioned above. Additionally, expression of enzymes involved in TAG synthesis including *GPAT* and *AGPAT* was also affected by altered expression of *SCD*1. Increase of UFAs including C16:1, C18:1 and C18:2 in the BMECs in response to overexpression of SCD1 may be a cumulative outcome of *ACACA* and *FAS* down-regulation together with up-regulation of *GPAT* and *AGPAT*, which is supported by the evidence that medium and long chain FA are potent inhibitors of *ACACA* in the mammary gland [18, 37]. It can be envisaged that *SCD*1 may corroborate with proteins which can inhibit the FA synthesis to maintain an appropriate FA synthesis and oxidation balance by altering its products. Studies have provided evidence that overexpression *of SCD*1 in CHO cells can lead to a significant increase in TAG synthesis while decreasing FA oxidation [38, 39]. Moreover, down-regulation of *SCD*1 can lead to decrease in expression of genes related to TAG synthesis and results in lower cellular TAG contents in 3 T3-L1 preadipocytes [36], as observed in the present study. Moreover, down-regulation of gene expression in the mammary gland has been reported to contribute to the overall decrease in milk fat synthesis [40].

## Conclusion

In conclusion, this study indicated that *SCD*1 plays an important role in FA and TAG synthesis and can directly or indirectly affect fat metabolic pathways in BMECs. Our study provides mechanistic insights on transcriptional regulation of *SCD*1 to alter FA and TAG synthesis through directly or indirectly mediating biosynthesis and metabolic pathways in BMECs. We provide the preliminary findings regarding the engineering of FA contents in buffalo milk through *SCD*1 signaling; however, further studies are required to fully understand the mechanism to elaborate regulatory gene networks.

## Methods

### Animals and sampling

Indigenous Holstein cows and purebred Mediterranean Buffaloes (*Bubalus bubalis*) imported from Italy in 2015 (by Guangxi HuaXu Buffalo Biotechnology co. LTD) were used for this study. All animals received humane care as outlined in the Guide for the Care and Use of Experimental Animals of the National Institutes of Health. Milk samples were collected from 30 Buffalos or Holstein cows in their 1st or 2nd parity and 100~150 days in lactation. We collected 30 samples from each species in this study and each milk sample were collected in triplicate until the composition (lactose, fat, protein, total solid and solid not fat) was determined by a milk analyzer (MilkoScan FT120, Denmark) immediately after collection. Milk samples were collected during the daily production without any needless hurt to the animal. BMECs of 2 lactigenous buffalo were collected from a slaughter house of Nanning (China) under sterile conditions. Instantaneous high-voltage shock was used to euthanasia the buffalo according to the Regulations for the Administration of Affairs Concerning Experimental Animals.

### Milk fat extraction and gas chromatography analysis

Milk fat extraction and gas chromatography analysis were performed following the method reported by Mele [41]. In brief, two grams of milk sample was mixed with 0.4 mL of ammonia 25%, 1 mL of ethyl alcohol 95%, and 5 mL of hexane, vortexed and centrifuged at 1600×g at 4 °C. The upper layer was collected, and a second extraction with 1 mL of ethyl alcohol 95% and 5 mL of hexane was performed. A third extraction was made by using 5 mL of hexane. The extracted fat was dried, weighed, and finally dissolved in hexane. FA composition was determined by gas chromatography using a Shimadzu GC-2014C (Kyoto, Japan) gas-chromatograph equipped with an FID and a capillary column (Agilent DB23, California, USA; 30 m × 0.32 mm i.d.; film thickness 0.25 μm). Injection ported 230 °C and detector 280 °C. The column was kept at 180 °C for 5 min and heated up to 230 °C at 3 °C min^− 1^. The carrier gas is kept in high-purity nitrogen and the injection volume is 1 μL. Individual FA methyl esters were identified by comparing them to a standard mixture of 37 Component FAME Mix (Supelco, Bellefonte, PA). The standards of PUFA-2, nonconjugated C18:2 isomer mixture, individuals cis-5,8,11,14,17 C20:5, cis-4,7,10,13,16,19 C22:6 (Supelco), cis-6,9,12 C18:3 and cis-9,12,15 C18:3 (Matreya Inc., Pleasant Gap, PA) were used to identify polyunsaturated FA. The identification of C18:1 isomer was based on commercial standard mixtures (Supelco) and published isomeric profiles (Wolff and Bayard, 1995). A nonadecanoic acid was used as an internal standard to increase the veracity of the peak normalization. For all studied FA, the coefficient of variation [(SD/mean) × 100] was < 3.5%, suggesting good repeatability of GC data. All milk FA compositions were expressed as g per 100 g of fat.

### Total RNA isolation

The total RNA in milk fat globules (MFG) was used for gene expression analysis as reported previously [42]. Milk samples were collected in the morning and mRNA was extracted immediately. The whole extraction process was performed at 4 °C (from the milk collection to the RNA extraction) and completed within 2 h to improve the quality of mRNA. Milk samples were centrifuged at 2000×g for 10 min at 4 °C to isolate milk fat. The supernatant fat layer was separated and 500 μL fat was mixed with 1.5 mL of TRIzol LS solution (Invitrogen Life Technologies Inc., Carlsbad, CA). All the procedures were carried out at 4 °C. RNA was dissolved in 30 μL of RNase-free water and stored at − 80 °C. We further performed the agarose gel electrophoresis analysis and only the mRNA samples with a low 5 s band were selected for the following experiment.

### Single-Strand cDNA synthesis

RNA purity was evaluated through absorbance readings (ratio of A260/A230 and A260/A280) by using a NanoDrop ND-2000 spectrophotometer (Thermo Fisher Scientific, Waltham, MA). Before first-strand cDNA synthesis, contaminated genomic DNA was removed by DNase treatment. First-strand cDNA was synthesized using RevertAid First Strand cDNA Synthesis Kit (K1622, Thermo Fisher Scientific, Waltham, MA). The single-strand cDNA obtained was stored at − 20 °C.

### Primer designing and qRT-PCR

Primers of the selected genes were designed by using the oligo 7 software (Table 5). The expressions of selected genes were quantified by using SYBR Green dye (4,913,914,001, ROCHE) and fluorescence data were acquired using RT-PCR instrument (ABI 7500). A 20 μL mixture was performed in each run as follows: 1 μL cDNA, 8 μL H_2_O, 10 μL Faststart Universal SYBR Green Master (ROX), and 0.5 uM aliquots of both forward and reverse primers. The thermal cycling profile started with a 3-min dwell temperature of 94 °C, followed by 40 cycles of 30 s at 94 °C, 30 s at the primer specific annealing temperature (60 °C), 30 s at 72 °C, and a final step during which fluorescence was acquired. After 40 cycles, a melting curve was generated by temperature increments of 0.1 °C starting three 3 times, and relative gene expression was calculated using the 2^-△△Ct^ method using *GAPDH* as a reference gene as reported previously [43].

**Table 5:** Primers used for real-time quantitative PCR

Target genes	Primer Sequences(5′~ 3′)	Tm (°C)	GenBank Accession No.
SCD1	F: TCCTGTTGTTGTGCTTCATCC R: GGCATAACGGAATAAGGTGGC	59	AY241933
FAS	F: GCAAAGTGGTCATTCAGGTACG R: CCCAGTGATGATGTAGCTCTTG	60	KM980092.1
PPARG	F: CAGTGTCTGCAAGGACCTCA R: GTAAAAGGCATGGGAGTGAT	59	HG270143.1
SREBP1	F: CTGACGACCGTGAAAACAGA R: AGACGGCAGATTTATTCAACTT	58	KU517672.1
GPAT	F: GCAGGTTTATCCAGTATGGCATT R: GGACTGATATCTTCCTGATCATCTTG	59	AY515690.1
AGPAT6	F: AAGCAAGTTGCCCATCCTCA R: AAACTGTGGCTCCAATTTCGA	59	DY208485
ACACA	F: TCCTGCTGCTATCGCTACTCCA R: CGCACTCACATAACCAACCAT	61	DQ773054.1
GAPDH	F: TGGAAAGGCCATCACCATCT R:CCCACTTGATGTTGGCAG	60	NM001034034.1

### Construction of Lentiviral vector and synthesis of siRNA

The SCD1 gene was cloned from buffalo mammary epithelial cells according to the SCD1 sequence available in GenBank (No. AY241933) and inserted into lentiviral vector. The siRNA targeting SCD1 gene were designed and synthesized by Gene Pharma (Shanghai, China) with a control sequence (Table 6). Lentiviral vectors containing the SCD1 gene and the siRNA with negative control (NC) were constructed by Gene Pharma (Shanghai, China). The lentiviral vectors were packaged and propagated in 293 T cell line with the packaging plasmid (ΔNRF) and envelope plasmid encoding the vesicular stomatitis Virus-G glycoprotein (VSVG).

**Table 6:** siRNA sequence target SCD1

siRNA target SCD1	Sequence (5′ to 3′)
siRNA-1	GCCCAAGCUUGAGUAUGUUTT
	AACAUACUCAAGCUUGGGCTT
siRNA-2	GCCCUAUAUGGGAUCACAUTT
	AUGUGAUCCCAUAUAGGGCTT
siRNA-3	GGAGUCACCGAACCUACAATT
	UUGUAGGUUCGGUGACUCCTT
siRNA-NC	UUCUCCGAACGUGUCACGUTT
	ACGUGACACGUUCGGAGAATT

### Cell culture

The BMECs were cultured and purified as reported previously [44, 45]. Briefly, during lactation fresh tissue blocks from buffalo were obtained and washed 3 times and the acinus portion was extracted from mammary gland tissue and transferred into high-resistance PBS (containing 400 IU mL^− 1^ of penicillin and 400 IU mL^− 1^ of streptomycin). Then tissue pieces were placed in culture dishes on a clean bench, cut into 1 to 2 mm pieces and tiled on the bottom of the culture dish and cultured in the incubator for 4 h. Then they were inverted and cultured in the upright position overnight. The epithelial cells started to grow after about 12 days and epithelial cells were isolated by using trypsin digestion combined with a cell adherence speed method. The purification procedure was performed 3 times and the BMECs at 3 to 4 generation in the subculture were used for the following studies;

### Infection and transfection

Transfection of the lentiviral vectors was carried out by using the Lipofectamine™ 2000 (Invitrogen, USA). 100 μM lentiviral vectors containing siRNA or SCD1 gene were used in each transfection and the transfected confluent cells were harvested for qRT-PCR analysis, 24 h post transfection.

### Statistical analysis

All experiments were repeated three times. Results were expressed as mean ± standard error (SEM). Statistical analysis was performed by using Student’s t-test and analysis of variance (ANOVA) with DUNCAN’s Multiple Range Test (DMR) in SPSS 17.0 software (IBM Corp., Armonk, NY). Differences of *P* < 0.05 were considered to be significant.

## Data Availability

The dates generated and analyzed during this study are included in this paper. Additional datasets used and/or analyzed during the current study are available from the corresponding author on reasonable request.

## Abbreviations

ACACA: Acetyl-coenzyme A carboxylase alpha
AGPAT: Acyl glycerol phosphate acyl transferase
DGAT: Diacylglycerol acyltransferase
FAS: Fatty acid synthase
GPAT: Glycerol-3 phosphate acyl transferase
MUFA: Monounsaturated fatty acid
PPARγ: Peroxisome proliferator activated receptor gamma
PUFA: Polyunsaturated fatty acid
SCD: Stearoyl-CoA desaturase
SCFA: Short chain fatty acid
SFA: Saturated fatty acid
UFA: Unsaturated fatty acid
